# A commentary on ‘Impact of lymphadenectomy extent on immunotherapy efficacy in postresectional recurred non-small cell lung cancer: a multi-institutional retrospective cohort study’

**DOI:** 10.1097/JS9.0000000000001347

**Published:** 2024-03-12

**Authors:** Chengcheng Zhang, Jingru Qin, Xiaoxue Zhao, Lu Yang, Zhongqi Wang

**Affiliations:** Department of Medical Oncology, Longhua Hospital affiliated to Shanghai University of Traditional Chinese Medicine, Shanghai, People’s Republic of China

*Dear Editor,*

It was with great interest that we read the article published in the *International Journal of Surgery*. Deng *et al*.^1^ conducted a retrospective cohort study on non-small cell lung cancer (NSCLC) patients with anti-PD-1 immunotherapy for recurrence. The findings showed that lower immunotherapy efficacy in recurrent NSCLC was correlated with an elevated dissected lymph node (DLN) count (cutoff: 16), a correlation that was particularly strong in the immunotherapy alone subgroup. The ratios of CD8+Tcm in lymph nodes may also affect the effectiveness of immunotherapy.

With around 85% of people having NSCLC, lung cancer is the primary cause of cancer-related death globally^2^. Radiation surgical resection is still the gold standard of therapy for NSCLC that is resectable at an early stage^3^. The 5-year survival rate following resection, however, is only 50–60%. In addition to being utilized therapeutically to remove metastatic disease for loco-regional control, lymph node dissection (LND) is also used to establish the best nodal staging for adjuvant therapy or prognostic prediction^4^. However, insufficient LND could be very detrimental since it could result in stage migration or residual metastatic disease, which is a sign of how well the NSCLC surgery went. One of the main purposes of LND is to confirm the number of LNs studied, and the quantity is related to the prognosis of different types of cancer. Nevertheless, there hasn’t been enough discussion of how the quantity of LNs investigated in NSCLC affects prognosis.

This multi-institutional retrospective study aims to demonstrate the immunological implications of LN dissection at the quantitative level for NSCLC patients with validation from multicenters and propose a novel concept of ʻimmunotherapy-drivenʼ LN dissection strategy. And it well expounded the advantages and limitations. This innovative strategy offers insightful direction and motivation for the ongoing advancement of this field of study. But there remained some things to be concerned about. First, this was a retrospective study; therefore, unobserved potential confounders could not be resolved. Second, the study only included patients from China, which may cause selection bias. Finally, this study was conducted in four centers with a small sample size. Therefore, further study required large sample sizes.

More detailed and multicenter research is required to confirm this result. Furthermore, deeper prospective research, clinical trials, and examination of indications and risk factors in various patient subgroups are required to identify the best course of action. Notably, mediastinal LND (MLND) is an essential component of lung cancer surgery. The number of examined mediastinal lymph node (mLN) dissections also affects the patient’s prognosis. Kamigaichi *et al*.^5^ showed that examining at least 3 mLNs had a survival benefit in patients with surgically resected clinical stage I–II, N0 NSCLC. Subgroup analysis and long-term follow-up are required if enough cases are added in the future to undertake time-series analysis.

## Ethical approval

This manuscript is a comment. Don’t need ethical approval.

## Consent

This manuscript is a comment. Don’t need patient consent.

## Sources of funding

This study was supported in part by the Shanghai Municipal Health Commission, Health Youth Talent Project (2022YQ028), Shanghai Hospital Development Center (SHDC2020CR4050), Shanghai Shenkang Hospital Development Center (SHDC12020123), and Shanghai Medical Innovation Research Project of ‘Science and Technology Innovation Action Plan’ (No. 20Y21902300).

## Author contribution

C.Z. and J.Q.: study concept or design, data collection, data analysis or interpretation, and writing the paper; X.Z. and L.Y.: data collection; Z.W.: study concept or design, writing, and revising the paper.

## Conflicts of interest disclosure

This manuscript is a comment without conflicts of interest.

## Research registration unique identifying number (UIN)

This manuscript is a comment. Don’t need UIN.

## Guarantor

Zhongqi Wang.

## Data availability statement

This manuscript is a comment. Don’t need a Data availability statement. However, all the data from the current study are publicly available.

## Provenance and peer review

This manuscript is a comment without being invited.
